# Overexpression of a *S*-Adenosylmethionine Decarboxylase from Sugar Beet M14 Increased *Araidopsis* Salt Tolerance

**DOI:** 10.3390/ijms20081990

**Published:** 2019-04-23

**Authors:** Meichao Ji, Kun Wang, Lin Wang, Sixue Chen, Haiying Li, Chunquan Ma, Yuguang Wang

**Affiliations:** 1 Engineering Research Center of Agricultural Microbiology Technology, Ministry of Education, Heilongjiang University, Harbin 150080, China; jimeichao25@gmail.com (M.J.); laissezfairewk@gmail.com (K.W.); wanglin1995@gmail.com (L.W.); schen@ufl.edu (S.C.); 1999020@hlju.edu.cn (H.L.); 2 Key Laboratory of Molecular Biology, College of Heilongjiang Province, College of Life Sciences, Heilongjiang University, Harbin 150080, China; 3 Department of Biology, Genetics Institute, Plant Molecular and Cellular Biology Program, University of Florida, Gainesville, FL 32610, USA; 4 Key Laboratory of Sugar Beet Genetic Breeding of Heilongjiang Province, Heilongjiang University, Harbin 150080, China

**Keywords:** sugar beet, salt stress, *S*-adenosylmethionine decarboxylase, ROS, antioxidant enzyme

## Abstract

Polyamines play an important role in plant growth and development, and response to abiotic stresses. Previously, differentially expressed proteins in sugar beet M14 (*Bv*M14) under salt stress were identified by iTRAQ-based quantitative proteomics. One of the proteins was an S-adenosylmethionine decarboxylase (SAMDC), a key rate-limiting enzyme involved in the biosynthesis of polyamines. In this study, the *BvM14-SAMDC* gene was cloned from the sugar beet M14. The full-length *BvM14-SAMDC* was 1960 bp, and its ORF contained 1119 bp encoding the SAMDC of 372 amino acids. In addition, we expressed the coding sequence of *BvM14-SAMDC* in *Escherichia coli* and purified the ~40 kD BvM14-SAMDC with high enzymatic activity. Quantitative real-time PCR analysis revealed that the *BvM14-SAMDC* was up-regulated in the *Bv*M14 roots and leaves under salt stress. To investigate the functions of the *BvM14-SAMDC*, it was constitutively expressed in *Arabidopsis thaliana*. The transgenic plants exhibited greater salt stress tolerance, as evidenced by longer root length and higher fresh weight and chlorophyll content than wild type (WT) under salt treatment. The levels of spermidine (Spd) and spermin (Spm) concentrations were increased in the transgenic plants as compared with the WT. Furthermore, the overexpression plants showed higher activities of antioxidant enzymes and decreased cell membrane damage. Compared with WT, they also had low expression levels of *RbohD* and *RbohF*, which are involved in reactive oxygen species (ROS) production. Together, these results suggest that the BvM14-SAMDC mediated biosynthesis of Spm and Spd contributes to plant salt stress tolerance through enhancing antioxidant enzymes and decreasing ROS generation.

## 1. Introduction

Plant growth and development was frequently affected by adverse environment factors (e.g., cold, salt, alkali and drought), and salt stress is a major factor limiting crop production [1]. When plants are exposed to salinity environment, the processes of protein synthesis and photosynthesis can be inhibited by excessive accumulation of salt in plants [2]. Furthermore, under salt stress conditions, excessive reactive oxygen species (ROS) were accumulated in the plant, and high levels of ROS can cause membrane lipid peroxidation and metabolic perturbation [3]. In order to survive the salinity, plants have evolved a series of complex tolerance mechanisms to prevent damage caused by salt stress from morphological, physiological and biochemical levels [4,5,6]. 

Polyamines (PAs) play an important role in regulating plant morphology, growth, embryonic development and response to stress conditions [7,8,9]. In higher plants, putrescine (Put), spermine (Spm) and spermidine (Spd) are the main types of polyamines. In the process of PA synthesis, Put is firstly synthesized from arginine catalyzed by arginine decarboxylase, agmatine iminohydrolase and N-carbamoylputrescine amidohydrolase. Then through spermidine synthase and spermine synthase, Put is converted to Spm and Spd with decarboxylated S-adenosylmethionine (dcSAM) as a donor [10,11]. dcSAM is synthesized from the decarboxylation of S-adenosylmethionine (SAM) in a reaction catalyzed by SAM decarboxylase (SAMDC) [12].

Previous studies reported that polyamines were involved in plant responses to abiotic stresses such as cold, drought and salinity [13,14]. Several enzymes involved in polyamine synthesis were shown to be important in plant stress tolerance [15,16]. For example, transgenic plants overexpression of *SAM* synthase *(SAMS)* from tomato showed enhanced alkali stress tolerance and maintained nutrient acquisition under the alkali stress [17]. Similarly, overexpressing a sugar beet *BvM14-SAMS2* in *Arabidopsis* increased salt and H_2_O_2_ tolerance of the plants [18]. SAMDC is a key enzyme in the synthesis of Spm and Spd. Down-regulation of *SAMDC* expression could decrease the Spm and Spd contents and lead to sensitivity to biotic and abiotic stresses [19,20]. In addition, overexpression of *SAMDC* was shown to play an important role in improving plant stress tolerance [21]. However, the mechanisms underlying the SAMDC regulation of plant tolerance to biotic and abiotic stresses are still to be investigated. 

Sugar beet monosomic addition line M14 was obtained through hybridization between sugar beet (*Beta vulgaris* L.) and wild sugar beet (*Beta corolliflora* Zoss.) [22,23]. The M14 line exhibits several interesting characteristics, such as salt and cold tolerance, as well as apomixes. These traits may be attributed to the retention of the 9th wild sugar beet chromosome in the M14 [24]. Previously, we used iTRAQ-based proteomics to profile protein changes in the M14 line under salt stress [25] and found a *BvM14-SAMDC* was strongly induced by salt stress. In the present study, we aim to gain insight into the mechanisms of BvM14-SAMDC mediated salt stress tolerance by overexpressing the *BvM14-SAMDC* in *Arabidopsis.* To understand the *BvM14-SAMDC* functions, polyamine contents, antioxidant enzyme activities and expression levels of antioxidant and ROS related genes were analyzed in the transgenic plants. Our results demonstrated that BvM14-SAMDC enhances salt stress tolerance through elevating the antioxidant system and suppressing ROS generation.

## 2. Results

### 2.1. Isolation of BvM14- SAMDC and Sequence Analysis

In order to acquire the sequence information of *BvM14-SAMDC*, a unigene with significant homology to plant *SAMDC* was found in our sugar beet transcriptome data set. Based on the unigene sequence and a RACE method, a full-length cDNA of *BvM14-SAMDC* were amplified by RT-PCR. The full-length *BvM14-SAMDC* is comprised of 1,960 bp with an open reading frame of 1119 bp nucleotides (Figure S1). The putative BvM14-SAMDC protein contains 372 amino acids with a predicted molecular mass of 40.7 kDa and a pI of 4.65. A large number of plant SAMDC homologs are present in the NCBI non-redundant database by BLASTP search using the BvM14-SAMDC sequence as a query. The BvM14-SAMDC protein shared 69–85% amino acid sequence identity with other plant SAMDCs.

### 2.2. Prokaryotic Expression and Enzymatic Activity Assay of Recombinant BvM14-SAMDC 

**Figure 1:**
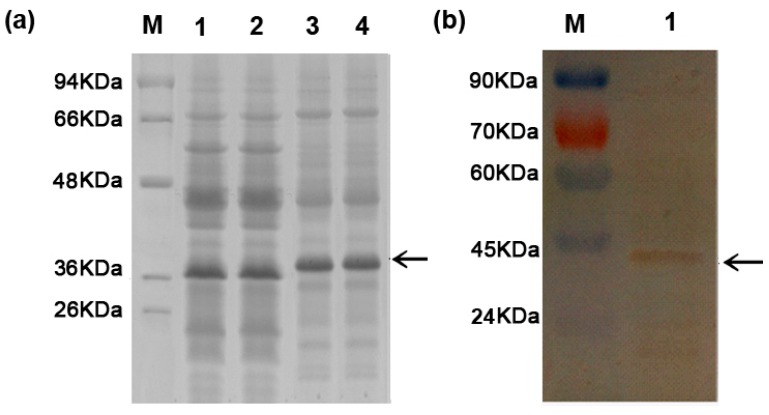
*Ecoli* expression of the recombinant sugar beet M14 S-adenosylmethionine decarboxylase (BvM14-SAMDC) protein. (**a**) Sodium dodecyl sulfate polyacrylamide gel electrophoresis (SDS-PAGE) analysis of bacterial expression of BvM14-SAMDC. Total soluble protein fractions with the empty vector after 4 h isopropyl β-d-thiogalactoside (IPTG) induction (lanes 1 and 2); and total soluble protein fractions with pET28a-*BvM14-SAMDC* after 4 h IPTG induction (lanes 3 and 4). (**b**) Immunoblot identification of the purified recombinant BvM14-SAMDC using anti-His antibody. Arrows indicate the BvM14-SAMDC position.

To test whether the isolated *BvM14-SAMDC* codes for a functional protein, the open reading frame was cloned under the control of the T7 promoter in a pET28a vector, and transformed into *E.coli* cells. Following induction with 0.1 mM IPTG, a ~40 kDa protein band could be observed on an SDS gel of protein extracts from the *E.coli* cells transformed with *BvM14-SAMDC*, but not with the empty vector (Figure 1a). After purification of the protein extract through a nickel column, the ~40 kDa BvM14-SAMDC could be identified on Western with an anti-His-tag antibody (Figure 1b). The purified protein is active with a specific activity of 2.11 units/mg. This result demonstrates that the *BvM14-SAMDC* gene encodes the right protein with SAMDC enzymatic activity.

### 2.3. Expression Patterns of BvM14-SAMDC under Salt Stress

**Figure 2:**
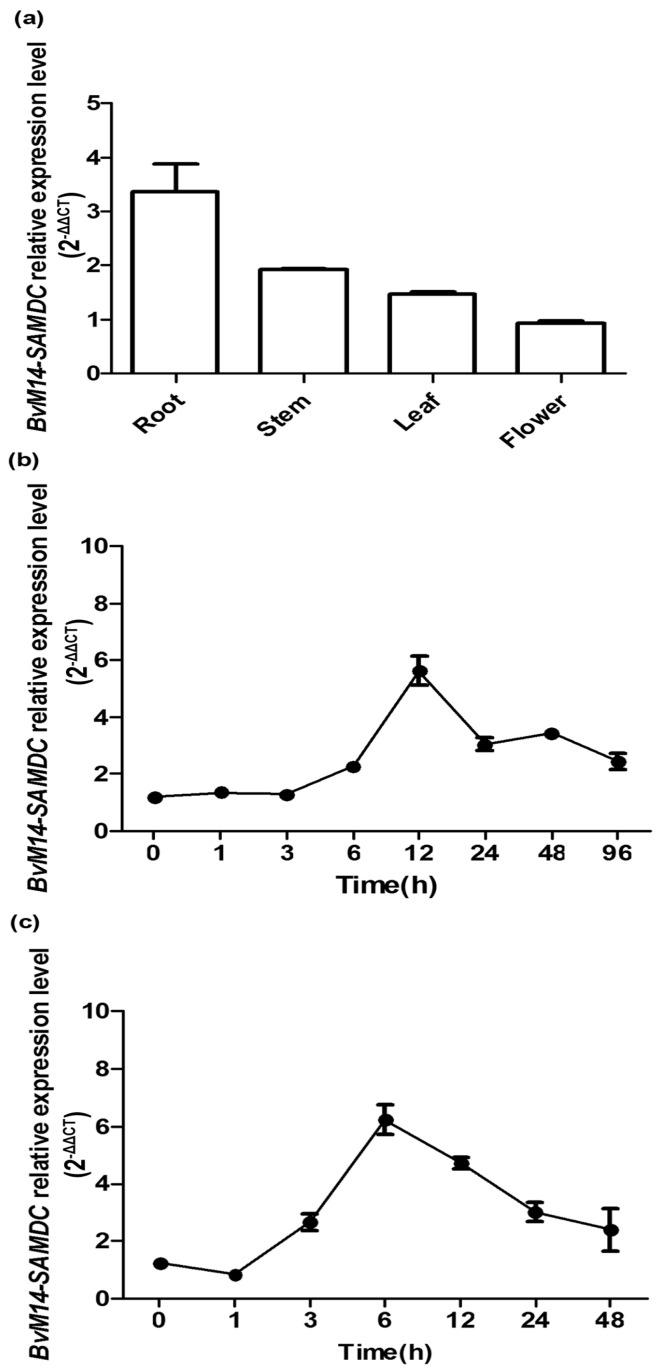
*BvM14-SAMDC* expression patterns in different organs and in response to salt stress treatments. (**a**) BvM14-SAMDC expression profiles in different organs. (**b**) leaves and (**c**) roots of the M14 plants treated with 400 mM NaCl for different time periods. Data are the means of three biological replicates with standard deviation (SD) bars.

To analyze tissue expression patterns of *BvM14-SAMDC*, real-time quantitative PCR was conducted using RNA extracted from in roots, stems, flowers and leaves of the M14 line. As shown in Figure 2a, *BvM14-SAMDC* was ubiquitously expressed in different organs, with roots showing higher levels than other organs (Figure 2a). Furthermore, to determine the response of *BvM14-SAMDC* to salt stress, the expression patterns of *BvM14-SAMDC* were analyzed in roots and leaves under 400 mM NaCl treatment and sampled at different time points. After salt stress, the increasing *BvM14-SAMDC* transcript level appeared much earlier in the M14 roots than in leaves (Figure 2b,c). The maximum levels of *BvM14-SAMDC* expression were observed at 6 and 12 h in roots and leaves, respectively, under salt stress. The results showed that *BvM14-SAMDC* transcription was significantly induced in roots and leaves by salt stress. Therefore, it is reasonable to speculate that *BvM14-SAMDC* participates in regulating plant salt stress tolerance.

### 2.4. Overexpression of BvM14-SAMDC Increased Salt Stress Tolerance in Arabidopsis

To study the functions of *BvM14-SAMDC* under salinity stress, we used *Agrobacterium tumefaciens* mediated transformation to produce transgenic Arabidopsis plants over-expressing the *BvM14-SAMDC* gene (Figure S2a). BLASTp analysis of BvM14-SAMDC protein sequences in the *Arabidopsis thaliana* database showed that the BvM14-SAMDC had the highest sequence similarity (77%) to an Arabidopsis SAMDC1 protein (AtSAMDC1). Therefore, a T-DNA insertion mutant of *AtSAMDC1* was selected and verified (Figure S2b,c). The expression level of *AtSAMDC1* was decreased by 72.5% in the *atsamdc1* mutant (KD) compared with WT, which was caused by the T-DNA insertion in the promoter region (Figure S2d,e). Furthermore, we transformed the *atsamdc1* mutant line with the *BvM14-SAMDC* overexpression construct to generate homozygous T3 complementation transgenic lines (CO1 and CO2) (Figure S2f).

**Figure 3:**
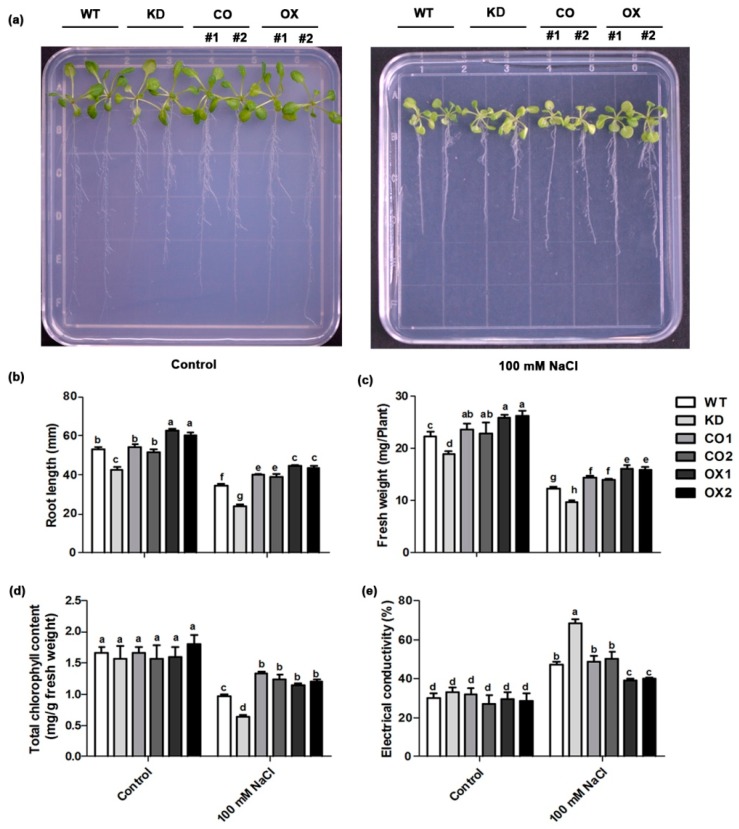
Effect of salt stress on seedling growth phenotype, root length, fresh weight, chlorophyll and electrical conductivity in wild type (WT), *BvM14-SAMDC*-overexpressed in wild type *Arabidopsis* (OX), *atsamdc1* mutant (KD) and transgenic *BvM14-SAMDC* in the mutant seedlings (CO) leaves. (**a**) 8-day-old WT and transgenic seedlings, grown on MS medium, were transferred to new MS solid agar plates supplemented with 0 or 100 mM NaCl, followed by growth for 10 days; (**b**) root length; (**c**) fresh weight; (**d**) chlorophyll level; and (**e**) electrical conductivity in control and 100 mM NaCl treated seedlings. Different letters indicate significantly different at *P* < 0.05. Three biological replicates were performed.

Following salt stress, plant growth was significantly affected in WT and all transgenic lines (Figure 3a). However, the root length, fresh weight and chlorophyll content were higher in transgenic plants overexpressing *BvM14-SAMDC* under salt stress compared with WT or *atsamdc1* (Figure 3b–e). Furthermore, we found *atsamdc1* were more sensitive to salt stress than the complementation lines (CO1 and CO2) or WT. Moreover, relative electrical conductivity was found to be much higher in the KD lines than the WT, complementation lines (CO1 and CO2) under salt stress conditions (Figure 3e). The relative electrical conductivity of KD was 1.44-fold higher than that of the WT under salt stress. Usually, the relative conductivity is an important index to evaluate cell membrane permeability. The lower value in the OX lines under salinity stress indicated that overexpressing *BvM14-SAMDC* could alleviate cell membrane damage in the plants. Collectively, these results demonstrated that *BvM14-SAMDC* is involved in regulating plants salt stress response, and plants overexpressing *BvM14-SAMDC* are more tolerant to salt stress.

### 2.5. Overexpression of BvM14-SAMDC Enhanced Antioxidative Activities in Arabidopsis

**Figure 4:**
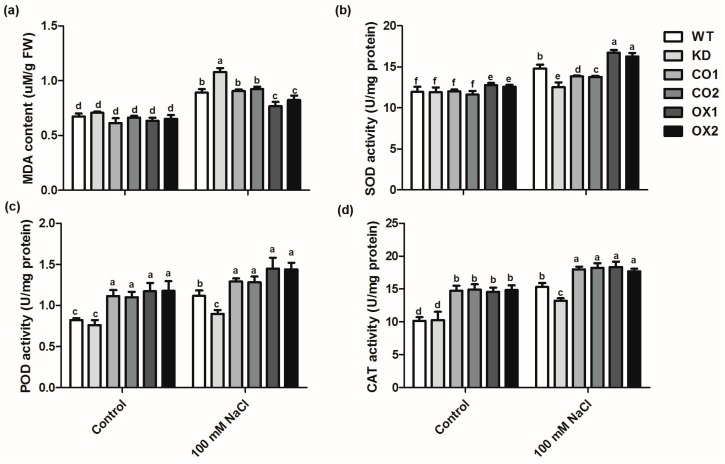
Effects of salt stress on antioxidant enzyme system in the leaves of wild type (WT), *BvM14-SAMDC*-overexpression in WT *Arabidopsis* (OX), *atsamdc1* mutant (KD) and transgenic *BvM14-SAMDC* in the mutant seedlings (CO). (**a**) leaf malondialdehyde (MDA) content; (**b**–**d**) antioxidant enzyme activities under control and salt stress (100 mM NaCl) conditions. One unit of CAT activity was defined as the amount of enzyme required for l μmol of H_2_O_2_ decomposed within 1 min. One unit of SOD activity was defined as the amount of enzyme required for inhibition of photochemical reduction of r-nitro blue tetrazolium chloride (NBT) by 50%. One unit of POD activity was defined as the amount of enzyme required for oxidation of 5 μmol of guaiacol within 1 min. Different letters indicate significantly different at *P* < 0.05. Three biological replicates were performed.

To determine how overexpression of *BvM14-SAMDC* affects the plant salt stress tolerance, malondialdehyde (MDA) content and antioxidant enzyme activities were determined in WT and the transgenic lines (Figure 4). Under stress conditions, MDA content was increased in all the genotypes (Figure 4a). However, the MDA level was dramatically higher in the KD mutant plants than transgenic or WT plants. In addition, *BvM14-SAMDC* overexpression plants (OX) showed lower MDA content than the WT and KD line under salt stress. Our results indicate that overexpressing *BvM14-SAMDC* could reduce the level of lipid peroxidation, which is tightly regulated by enzymes involved in ROS-detoxifying pathways. Therefore, we examined the activities of superoxide dismutase (SOD), catalase (CAT) and peroxidase (POD) (Figure 4b–d). After salt stress, the activities of SOD increased by 23.9%, 5.2% and 30.9% in WT, KD and OX1 lines, respectively. Moreover, the activities of SOD, CAT and POD in the leaves or roots of OX lines were significantly higher than WT or the mutant under control and salt stress conditions (Figure 4b–d and Figure S3). Overall, these results suggest that reduction of MDA can be attributed to the increased activities of ROS-detoxifying enzymes in the transgenic plants.

### 2.6. Overexpression of BvM14-SAMDC Enhanced PA Levels and PAO Activity in Arabidopsis

**Figure 5:**
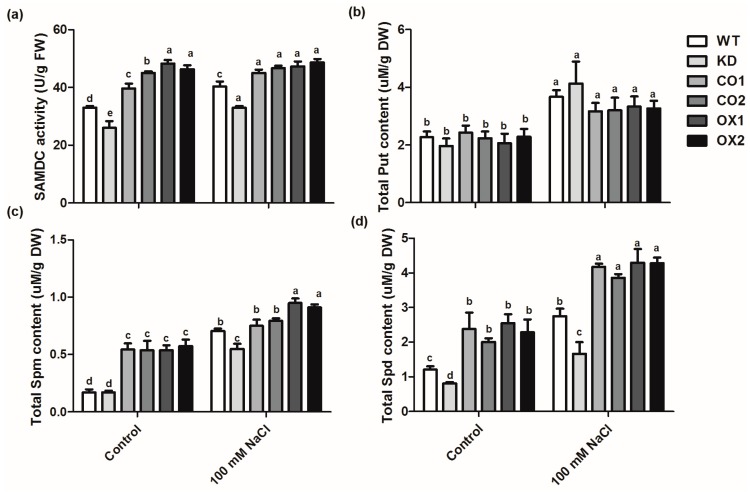
Effects of salt stress on polyamine metabolism in the leaves of wild type (WT), *BvM14-SAMDC*-overexpression in WT *Arabidopsis* (OX), *atsamdc1* mutant (KD) and transgenic *BvM14-SAMDC* in the mutant seedlings (CO). (**a**) SAMDC activity; (**b**) levels of putrescine (Put); (**c**) levels of spermine (Spm); and (**d**) levels of spermidine (Spd) under control and salt stress (100 mM NaCl) conditions. One unit of SAMDC activity was defined as the amount of enzyme required for catalyzing 2 μmol SAM within 1 min. Different letters indicate significantly different at *P* < 0.05. Three biological replicates were performed.

As to the overall SAMDC activities in *Arabidopsis*, the SAMDC activity of mutant line (KD) was 21.2% and 18.1% lower than that of WT under control and salt stress conditions, respectively. In contrast, the complementation line (CO) exhibited higher SAMDC activity than the wild type (Figure 5a). As expected, the SAMDC activity in OX1 line was 1.46 times higher than that of WT under control conditions. Since SAMDC plays an important role in PA synthesis, polyamines were measured in the transgenic plants in comparison with WT and the mutant lines (Figure 5b–d). Our result indicates that the Put level showed no difference between the WT and transgenic plants, but the Spd and Spm levels were significantly elevated in the transgenic plants. Furthermore, Spd and Spm contents in the mutant line were dramatically lower than the transgenic or WT plants. Clearly, the synthesis of Spd and Spm was improved by overexpressing the *BvM14-SAMDC*. 

**Figure 6:**
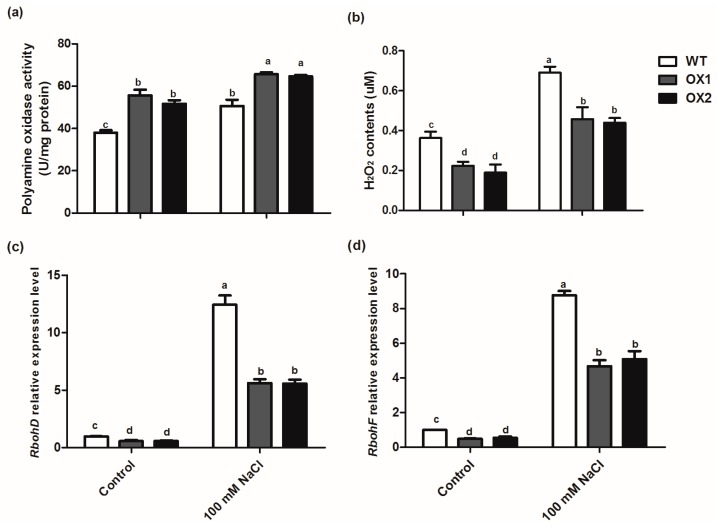
Effects of salt stress on polyamine oxidase (PAO) activity, H_2_O_2_ content and mRNA levels of *RbohD* and *RbohF* in the leaves of wild type (WT) and *BvM14-SAMDC*-overexpression in WT *Arabidopsis* (OX). (**a**) PAO activity; (**b**) H_2_O_2_ content; (**c**) mRNA levels of *RbohD*; and (**d**) mRNA levels of *RbohF* under control and salt stress (100 mM NaCl) conditions. One unit of PAO activity was defined as the amount of enzyme required for catalyzing 1 mmol of Spd or Spm oxidation within 1 min. Different letters indicate significantly different at *P* < 0.05. Three biological replicates were performed.

Previously, it is reported that PA played a key role in regulating H_2_O_2_ homeostasis through Spd and Spm catabolized by polyamine oxidase (PAO) to generate H_2_O_2_ in plants [11]. H_2_O_2_ is also an important signaling molecule to induce stress/defense responses. In the present study, higher activity of PAO was found in the transgenic plants than WT (Figure 6a and Figure S4a), and the H_2_O_2_ content was lower in the overexpression transgenic seedlings than in WT (Figure 6b). The generation of ROS may be attributed to the expressions of respiratory burst oxidase homolog genes (*RbohD* and *RbohF)*, which were significantly enhanced in the leaves or roots of WT under salt stress (Figure 6c,d and Figure S4b,c). Salt stress-enhanced the expression of *RbohD* and *RbohF* transcription was notably inhibited in the leaves and roots of two transgenic lines compared to WT plant (Figure 6c,d and Figure S4b,c). Therefore, these results indicate that PA could alleviate ROS damage in the transgenic plants by decreasing the expression of *RbohD* and *RbohF*.

## 3. Discussion

Plant PAs, namely spm, spd and put, play essential roles in many different developmental and physiological processes. In particular, plant PAs are involved in regulating plant resistance to various stresses [26,27]. As the key enzyme in the synthesis of Spm and Spd biosynthesis, SAMDCs play an important role in plant stress tolerance [28,29]. In our study, *BvM14-SAMDC* expression varied in different organs of sugar beet, and it might be associated with growth and developmental processes in the sugar beet M14. In addition, *BvM14-SAMDC* expression was significantly induced by salt stresses in roots and leaves. The increasing expression of *BvM14-SAMDC* appeared much earlier in the M14 roots than in leaves, suggesting that the response of PA metabolism to salt stress is rapid in roots compared with leaves. Usually, the photosynthetic pigment is closely associated with plant salinity tolerance [30]. The OX lines showed increases in the total chlorophyll content compared to WT and mutant plants under salt stress (Figure 3d). These results predict salt stress tolerance of the *BvM14-SAMDC* overexpression plant and the sensitivity of the mutant line to salt stress.

Other studies have demonstrated the functions of PAs as free radical scavengers under various stress conditions, and they inhibit lipid peroxidation [31,32]. Here the levels of diamine (Put) and triamine (Spd and Spm) were increased under salt stress conditions in wild type and the transgenic plants (Figure 5). This result is consistent with a previous report [33]. Treatment of *Solanum chilense* plants with 125 mM NaCl increased the production of Spm, involving in salt resistance in *S. chilense* [33]. Therefore, PAs play an important role in regulating plant salt stress tolerance. Moreover, the levels of Spd and Spm were much higher in the *BvM14-SAMDC* overexpression lines under both control and salt stress conditions, and there was no significant difference in put content between WT and the transgenic plants (Figure 5b). This situation will lead to a high ratio of (Spd + Spm)/Put in OX transgenic plans. It is reported that Spd and Spm play a key role in regulating the thylakoid membrane integrity, and a major function of put is to regulate cell membrane depolarization [34]. In addition, another study also showed that overexpression of a *SAMS* in tomato (another key enzyme in PA biosynthesis) caused a high ratio of (Spd + Spm)/Put and alkali stress tolerance through up-regulating *SlSAMDC* and *SlSPDS* [17]. They speculated that the high levels of *SlSAMDC* and *SlSPDS* could increase the conversion of Put to Spm and Spd. Therefore, the increased *BvM14-SAMDC* level in the transgenic plants may be involved in increasing the Spd and Spm levels for enhanced salt stress tolerance. 

**Figure 7:**
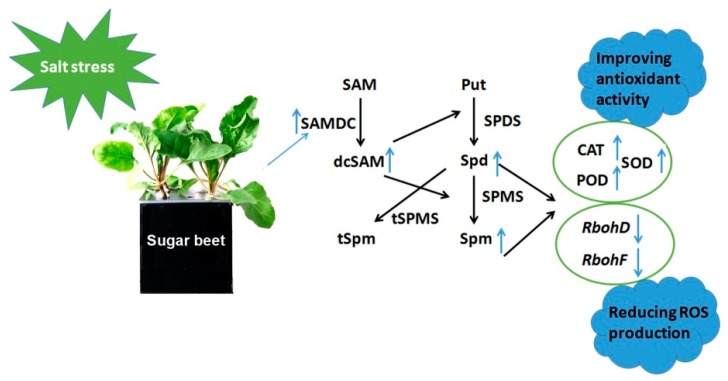
Overview diagram showing how the *Bv*M14-SAMDC functions in mediating plant salt stress tolerance. Salt stress can turn on the expression of BvM14-SAMDC, which plays an important role in the biosynthesis of PAs. PAs may inhibit the expression of ROS-biosynthetic enzymes and activate ROS-detoxifying enzymes, leading to reduced ROS levels and enhanced salt stress tolerance phenotype. SAMDC, S-adenosylmethionine decarboxylase; SPDS, spermidine synthase; SPMS, spermine synthase; tSPMS, thermospermine synthase; dcSAM, decarboxylated S-adenosylmethionine; Put, putrescine; SAM, S-adenosylmethionine; Spd, spermidine; Spm, spermine.

Previous studies proposed that SAMDC regulates the plant response to multiple abiotic stresses such as salinity, drought, cold and high temperature. These studies suggest that overexpression of SAMDC lead to polyamine accumulation, which can confer tolerance to both biotic and abiotic stresses in plants [35,36,37,38]. However, the mechanism of SAMDC gene improving plant stress resistance is not very clear. In the present study, compared to WT, higher activities of antioxidant enzymes were detected in the *BvM14-SAMDC* overexpression lines under both salt stress and control conditions. Furthermore, the MDA and H_2_O_2_ levels in the transgenic plants were significantly lower than in WT (Figure 4a and Figure 6b). It is reported that tobacco plants with decreased *SAMDC* expression showed low PA biosynthesis, and accumulated significantly more H_2_O_2_ [19]. In our study, although the elevated PAO activity was observed in the transgenic plants, H_2_O_2_ content was reduced (Figure 6b). We speculated that ROS production induced by PAO activity was effectively cleared by the ROS-detoxifying enzymes in the *BvM14-SAMDC* OX lines. In addition, the expression of the two plasma membrane localized NADPH-oxidases, *RbohD* and *RbohF* is often associated with ROS generation [39,40]. Therefore, our study showed that expression of *RbohD* and *RbohF* was dramatically enhanced in the WT under salt stress, and the increased expression was blocked by overexpressing the *BvM14-SAMDC* in the transgenic plants (Figure 6c,d and Figure S4b,c). These results show that *BvM14-SAMDC* regulates salt stress tolerance by improving antioxidant activity and reducing ROS production (Figure 7). Similarly, our previous study also found the activities of CAT and POD were higher in the sugar beet *SAMS2* overexpression plants than in WT, and the *SAMS2* plants had a low accumulation of H_2_O_2_ [18]. Whether SAMS2, like SAMDC, and improve plant stress resistance through reducing ROS production remains to be further studied.

## 4. Materials and Methods 

### 4.1. Plant Materials and Treatments

Sugar beet M14 was grown in a greenhouse of Heilongjiang University. After the M14 seeds were sown in vermiculite for 7 day, the seedlings were transferred to Hoagland nutrient solution with a 14 h light/10 h dark cycle, a 400 μmol m^−2^ s^−1^ light intensity, and 25 °C/21 °C day/night temperature. The 14 day old seedlings were subjected to 400 mM NaCl treatment. Sugar beet seedlings were also grown in pots with black soil from Heilongjiang University, and the organs of root, stem, leaf and flower were harvested after 3 months [41]. The *Arabidopsis* seeds were surface sterilized with the mixed solutions of NaClO (0.5%) and Triton X-100 (0.01%) for 8 min followed by washing with sterilized distilled water three times. The sterilized seeds were first incubated on Petri dishes containing Murashige and Skoog (MS) agar (0.8%) medium (containing 3% sucrose and 0.25% phyta-gel, pH 5.8) at 4 °C for 3 day before germination, and Petri dishes were sealed using parafilm. Then the seeds were germinated at 22 °C under 14 h at 300 μmol m^−2^ s^−1^ light intensity light and 10 h darkness, and parafilm was removed from the dishes. The 8-day-old seedlings were transferred to MS medium containing 100mM NaCl for salt treatment 10 day, and each Petri dish contained eight seedlings. 

### 4.2. Cloning the Ful- Length cDNA of BvM14-SAMDC Gene

A unigene of *BvM14-SAMDC* was used for designing primers (sense primer: 5′-TCTGCTGCT TACTCAAACTGCG- 3′ and antisense primer: 5′-TATTCCAACACGGGACACTGA- 3′) to amplify the full-length cDNA sequence of the *BvM14-SAMDC* gene using reverse transcription (RT)-PCR through a RACE method [41]. 

### 4.3. Real-time Quantitative PCR

Real-time quantitative PCR (qRT-PCR) was carried out to determine the gene expression in the M14 line and Arabidopsis. Total RNA was extracted using a Trizol reagent (Invitrogen, Carlsbad, CA, USA), and cDNAs were synthesized from 0.5 μg total RNA using SuperScript-RNase H-reverse transcriptase (Clontech, Foster, CA, USA). With 18S rRNA as the internal control, samples from different organs were amplified using a Bio-Rad Thermocycler system combined with SYBR-Green fluorescent dye (TaKaRa, Dalian, China) according to the manufacturer’s instructions. PCR reaction was carried out in 10 μL volumes using the following amplification protocol: 94 °C for 5 min; 94 °C for 35 s, 55 °C for 30 s, and 72 °C for 90 s; and 72 °C for 5 min, 45 cycles. The primers used for qRT-PCR analysis are listed in Table S1.

### 4.4. Heterologous Expression of BvM14-SAMDC in E.coli

The open reading frame (ORF) sequence of the *BvM14-SAMDC* gene was amplified with primers (5′-ATGACGGTTCCCATGGTTGG-3′ and 5′-ATTCATTTCTTCTTCTTTTTT-3′) and ligated into a pMD18-T vector. Then, the construction vector was cut with *EcoR* I and *Sal* I, the cut fragment was ligated with a pET28a carrier cut with *EcoR* I and *Sal* I. The construct was introduced into *E. coli* BL21 (DE3) for protein expression after induction with 0.5 mM IPTG. Cells were harvested after 4 h for total protein extracts from the induced *E. coli* transformed with pET28a-*BvM14-SAMDC*. The total protein extracts from the induced *E. coli* transformed with pET28a-*BvM14-SAMDC* were analyzed using 12 % SDS-PAGE and detected with anti-histidine monoclonal antibody linked to horseradish peroxidase (1:10,000) according to the manufacturer’s instructions (Pierce, Carlsbad, CA, USA). 

### 4.5. Arabidopsis Thaliana Mutant Screening

*Arabidopsis* T-DNA insertion lines were identified for *AtSAMDC1* mutation by genotyping PCR. Specific primers for the left and right borders of the T-DNA and for *AtSAMDC1* (F1: 5-ATTGAAGACGTTCGTCCAAAC-3, R1: 5-CTCGCCTTGTTGTGTGAGCGACAG-3, T1: 5-TAGCATCTGAATTTCATAACCAATCTCGATACAC-3) were used to identify the mutant lines. PCR reaction was carried out in 30 μL volumes using the following amplification protocol: 94 °C for 5 min; 94 °C for 30 s, 60 °C for 30 s, and 72 °C for 90 s; and 72 °C for 5min, 30 cycles.

### 4.6. Generation of 35S:BvM14-SAMDC Construct and Transformation of Arabidopsis

The ORF sequence of the *BvM14-SAMDC* was amplified using primers (5′-GTT CTTTTAAGCAATCTAG-3′ and 5′-ATTCATTTCTTCTTCTTTTTT-3′) and inserted into the pUCm-T vector. Then the construct was digested with using *EcoR* I and *BamH* I and inserted into a pCAMBIA1305 vector. *Agrobacterium tumefaciens* strain EHA105 containing pCAMBIA1305-*BvM14-SAMDC* was infiltrated into *Arabidopsis* using a floral dip method as previously described [42]. The presence of the transgene was confirmed by PCR and quantitative PCR.

### 4.7. Determination of Physiological Indicators

Chlorophyll (chl) content was measured according to our previous reported by homogenizing leaf samples (0.5 g) with 10 mL acetone (80% *v*/*v*) followed by centrifuging at 9000 g for 8 min. Absorbance was measured with a UV-visspectro photometer at 663 and 645 nm for chl a and chl b content, respectively. The activities of SOD and CAT were measured according to previously reported methods [17,43,44]. For the extraction of antioxidant enzyme, 1 g of sugar beet tissues were homogenised in 3 mL of chilled buffer containing 50 mM phosphate buffer (pH = 7.8), 2 mM EDTA, 1 mM DTT, 1 mM PMSF, 0.5% (*v*/*v*) Triton X-100 and 10% (*w*/*v*) PVP-40, and the homogenate was centrifuged at 20,000 g for 30 min. For measurement of SOD, 1mL of reaction mixture was prepared in 50 mM K-P buffer (pH 7.8) containing 2 μM riboflavin, 75 μM nitrotetrazolium blue (NBT), 100 μM EDTA, 13 mM DL-methionine and 60 μL of enzyme extract and the absorbance was taken at 560 nm. CAT enzymatic activity was calculated using the system reported by Aebi. The decline of H_2_O_2_ absorbance was determined in 2 mL of reaction buffer containing 100 mM K-P buffer (pH 7.0), 20 mM H_2_O_2_ and 20 μL enzyme extract. POD activity was measured by the increase in absorbance at 470 nm due to guaiacol oxidation

For MDA extraction, fresh leaf samples (0.5 g) were homogenized with 0.1% trichloroacetic acid (TCA). The homogenate was then centrifuged at 15,000 g for 10 min. An aliquot (1 mL) of the supernatant was mixed to 4 mL of 20% TCA prepared in 0.5% thiobarbituric acid (TBA) and incubated at 90 °C for 30 min in a shaking water bath. The reaction was stopped in an ice bath. The samples were then centrifuged at 10,000 g for 5 min, and the absorbance of the supernatant was measured at 532 nm.

For measurement of H_2_O_2__,_ fresh leaves (0.3 g) were frozen in liquid nitrogen and ground to powder in a mortar with a pestle, together with 5 mL of 5% TCA and 0.15 g activated charcoal. The mixture was centrifuged at 10,000 g for 20 min at 4 °C. The supernatant was adjusted to pH 8.4 with 17 M ammonia solution and then filtered. The filtrate was divided into aliquots of 1 mL. To one of these, the blank was added 8 μg of catalase and then kept at room temperatures for 10 min. To both aliquots with and without catalase, 1 mL of colorimetric reagent was added. The reaction solution was incubated for 10 min at 30 °C. Absorbance at 505 nm was determined spectrophotometrically. 

Polyamine levels were measured according to the procedure of Guo et al [9]. Leaves (0.5 g) were extracted in 5 mL of 5% (*v*/*v*) cold perchloric acid (PCA) and incubated on ice for 1 h. The homogenate was centrifuged at 20,000 g for 30 min. Aliquots (0.5 mL) of supernatant were mixed with 1 mL of 2 M NaOH and 7 mL of benzoyl chloride and incubated at 37 °C for 20 min in dark for benzoylation. The benzylated polyamines were extracted to diethyl ether and resuspended in 1 mL of mobile phase solution before HPLC analysis. Polyamine levels were calculated based on standard curves in combination with a recovery of the extraction procedure. The activity of SAMDC was assayed by HPLC using an enzyme activity standard curve [9].

Polyamine oxidase was extracted in 0.1 M phosphate buffer (PH 7.0), and the reaction was initiated and incubated at 30 °C for 30 min after addition of 20 mL of 20 mM Spd or Spd into the reaction mixture (3 mL) that consisted of 2.5 mL of 0.1 M phosphate buffer, 0.1 mL of horseradish peroxidase, 0.2 mL of coloring solution (25 mL *N*, *N*-dimethylaniline and 10 mg 4-aminoantipyrine were dissolved in 100 mL of 0.1 M phosphate buffer, pH 7.0) and 0.2 mL enzyme extract or inactivated enzyme (by heating the enzyme for 20 min in a boiling water bath). Absorbance at 550 nm was recorded.

## 5. Conclusions

In the present study, we discovered that overexpression of *BvM14-SAMDC* in *Arabidopsis* seedlings conferred salt stress tolerance. Furthermore, the overexpression lines (OX) reduced ROS accumulation, had decreased expression of *RbohD* and *RbohF*, and increased antioxidant enzyme activities. This work clearly showed that a key enzyme SAMDC in the biosynthesis of PAs can enhance salt stress tolerance through reducing ROS levels caused by inhibiting the expression of ROS-biosynthetic enzymes and activation of ROS-detoxifying enzymes. How does salt stress turn on the expression of *BvM14-SAMDC* is a very interesting question to be addressed in the near future.

## Supplementary Materials

Supplementary Materials can be found at https://www.mdpi.com/1422-0067/20/8/1990/s1.
