# Supplementary material for: Overexpression of a S-Adenosylmethionine Decarboxylase from Sugar Beet M14 Increased Araidopsis Salt Tolerance
Source: Int J Mol Sci. 2019 Apr 23;20(8):1990. doi: 10.3390/ijms20081990 (PMC6515516; doi:10.3390/ijms20081990)
Supplement: Supplementary file 1 [file ijms-20-01990-s001.pdf]

```
1       TACGCCAGCTATTTAGGTGACACTATAGGGGAAAGCTTGCATGCCTGCAGGTCGACTCTA
        GAGGATCTACTAGTCATATGGATTCTAATACGACTCACTATAGGGCAAGCAGTGGTATCA
        ACGCAGAGTACATGGGGATCGAAAAAAAGCCTGTTAATCCTTCGAAAAGCCGCCACTCTT
        ATTATTCATTCATCATCATCCTAATCTCTCTTCTCATCATATCCTCAATCTTGCGTCAAT
        TTTCTCTCTCCTAGGGTTTTCAATTCGCTGCAATTTTCCGACTTTTTCATTTTCAAGAAT
        CCAAGTGCTGGTTAGAGAAGCCGTTATTTTGGTTCATTCTTGATTTCCCCTTTTTCTTGT
        GAATGATCTAATGGAATCAAAAGGTGGTAAAGACGATTCTAGTAGTAGTAGTAGTAAATC
        CTTGTTCACCCCACTCGACTACGGCATTGAAGACGTTCGACCAAAAGGAGGAATCAAGAA
        ATTCAGATCTGCTGCTTACTCAAACTGCGCTCGCAAACCATCCTGATATTCCCCGTGCAC
        CGCCTGCTCGACCACGTGAAGTGGTTTAATTTAGTTTTAATTTTTGCATTAGAAATAACA
        GCTTTTGATTACAAGTTCCTTTCTTCTTCTGCTTCCCTCTTTCCTCTGCAACTCCGGTTC
661     CTCTTTGCGACTGTGAGACGTTCTTTTAAGCAATCTAGCTATGACGGTTCCCATGGTTGG
                                                 M  T  V  P  M  V  G
721     AGACAACAACGACAACAACATGACGATTTCTGCTATTGGATTTGAAGGGTATGAAAAGAG
          D  N  N  D  N  N  M  T  I  S  A  I  G  F  E  G  Y  E  K  S
781     GCTAGAGATAACGTTCTTTGAGCCTGGTATTTTTGTTGATGCTGACGGGAAGGGCCTCCG
          L  E  M  T  F  F  E  P  G  I  F  V  D  A  D  G  K  G  L  R
841     TGCTTTATCTAAGGATCAGTTGGATGAGATCCTTGGCCCTGCTGAGTGCACCATTGTTGA
          A  L  S  K  D  Q  L  D  E  I  L  G  P  A  E  C  T  I  V  D
901     CTCTCTTGCGAATGAAAGTGTAGACTCATATGTTCTATCCGAGTCTAGCCTCTTTGTGTA
          S  L  A  N  E  S  V  D  S  Y  V  L  S  E  S  S  L  F  V  Y
961     CTCATACAAGATCATCATAAAAACTTGTGGGACTACGAAGCTGCTCCTTGCAATCCCACC
          S  Y  K  I  I  M  K  T  C  G  T  T  K  L  L  L  A  I  P  P
1021    CATTTTGAAGTTGGCTGCAAGCCTATCCCTTGATATCAAAGCTGTCCGATACACTCGTGG
          I  L  K  L  A  A  S  L  S  L  D  I  K  A  V  R  Y  T  R  G
1081    CAGTTTTATTTTCCCAGGAGCTCAGTCTTTCCCTCACCGAAGCTTCTCTGAAGAAGTTGC
          S  F  I  F  P  G  A  Q  S  F  P  H  R  S  F  S  E  E  V  A
1141    TGTTCTTGATGGTTACTTTGGGAAGCTTGCTGCAGGCAGCAATGCCTTTGTGATGGGGAA
          V  L  D  G  Y  F  G  K  L  A  A  G  S  N  A  F  V  M  G  N
1201    TCCTTCTAAGCCCCAGAAATGGCATGTTTACTGTGCATCCGCCGAAACAACAGCCAGCTA
          P  S  K  P  Q  K  W  H  V  Y  C  A  S  A  E  T  T  A  S  Y
1261    TGATGACCCTGTTTATACTCTTGAGATGTGCATGACTGGGCTTAACAAGGAGAAAGCTTC
          D  D  P  V  Y  T  L  E  M  C  M  T  G  L  N  K  E  K  A  S
1321    AGTGTTCTTCAAAGCTCAATCTGACTCAGCTGCTGTGATGACTGAAAGCTCTGGAATCCG
          V  F  F  K  A  Q  S  D  S  A  A  V  M  T  E  S  S  G  I  R
1381    TAAGATCCTTCCAGACTCAGCGATTTGTGACTTTGATTTTGAACCTTGTGGTTATTCAAT
          K  I  L  P  D  S  A  I  C  D  F  D  F  E  P  C  G  Y  S  M
1441    GAACGCTATTGAAGGACCTGCTGTCTCTACCATCCACATAACTCCAGAAGATGGTTTTAG
          N  A  I  E  G  P  A  V  S  T  I  H  M  T  P  E  D  G  F  S
1501    TTACGCGAGTTTTGAAGCTGTGGGCTATGACCTTAAGATGATTGATTTGAACCAGCTGGT
          Y  A  S  F  E  A  V  G  Y  D  L  K  M  I  D  L  N  Q  L  V
1561    AGAGAGGGTCCTCGCTTGCTTTGAACCGAGTGAGTTCAGTATAGCTATTAATGCTGATAT
          E  S  V  L  A  C  F  E  P  S  E  F  S  M  A  I  N  A  D  I
1621    TGCTGCCTATCCAAAGGAGCAAAACTGTACCGTGAACGCCAACGGTTACAGTCGTGAAGA
          A  A  Y  P  K  E  Q  N  C  T  V  N  A  N  G  Y  S  R  E  E
1681    GGGTGGCATTGAAGAGCTTGGCTTTGGTGCTTCTGTATTCTACCAGAAGTTCTGCAAGGC
          G  G  I  E  E  L  G  F  G  A  S  V  F  Y  Q  K  F  C  K  A
1741    TACAACTATGATTGGTTTTACCAAGCCTGCTCTGAAATGCCTCTGGAAAGAGGAGAAAAA
          T  T  M  I  G  F  T  K  P  A  L  K  C  L  W  K  E  E  K  K
1801    AGAAGAAGAAATGAATTAGTACTTCATTTTATGTGTTCCATCTTTTTTTTTTTTTGAAAT
          E  E  E  M  N  *
1861    AAGAGTCCGGTCTTGAACCTGATTTTATGGTTATCTTTTCAGTGTCCCGTGTTGGAATAT
1921    TTTTATATTGTTATAATTTGAAGTGTATCCCGGATTATGC
```

Figure S1. Sequence analysis of a cDNA encoding a *BvM14-SAMDC* isolated from the monosomic addition line M14 roots. Nucleotide and deduced amino acid sequence of the *BvM14-SAMDC*.

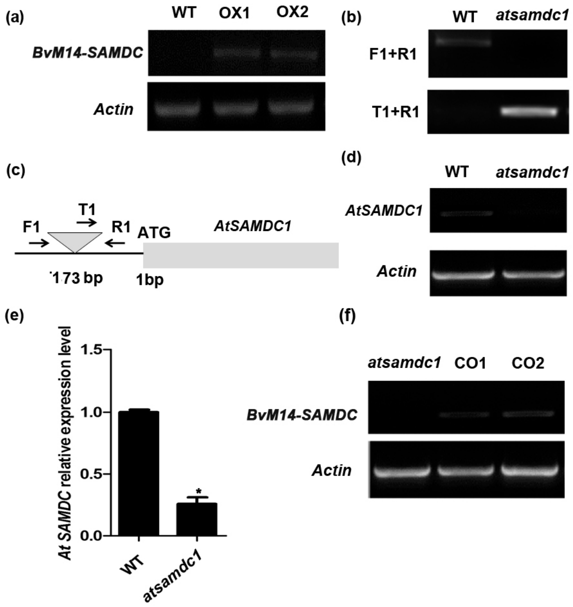

Figure S2. Identification of *atsamdc1* mutant and overexpression of *BvM14-SAMDC* in Arabidopsis. (a) RT-PCR analysis of the expression levels of the overexpressed *BvM14-SAMDC* (OX1 and OX2) in Arabidopsis; (b) PCR genotyping analysis of the T-DNA insertion in the *atsamdc1* mutant (KD); (c) Structure of the *AtSAMDC1* gene. The T-DNA insertion site was at 173 bp upstream of the start codon. The primers used to identify the T-DNA insertion were marked with arrows; (d) RT-PCR analysis of the expression levels of *AtSAMDC1* in *atsamdc1* mutant (complementation lines, CO) and wild type (WT); (e) Real-time PCR analysis of the expression levels of *AtSAMDC1* in the *atsamdc1* mutant; (f) RT-PCR analysis of the expression levels of the overexpressed *BvM14-SAMDC* (CO1 and CO2) in *atsamdc1* mutant. Asterisk (*) indicates significantly different at $P < 0.05$. Three biological replicates were performed. Please refer to supplementary materials for the primers used.

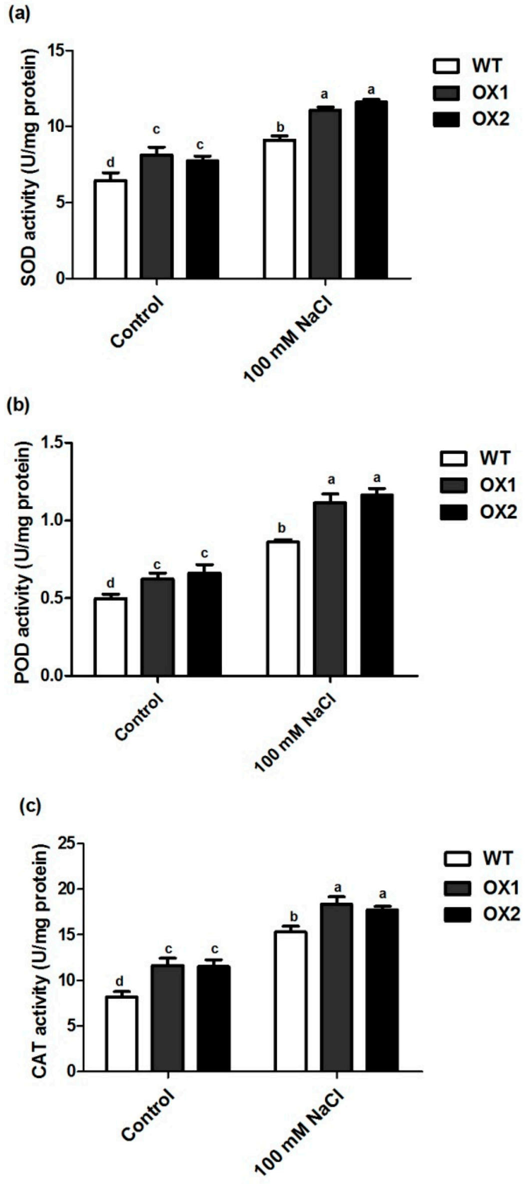

Figure S3. Effects of salt stress on antioxidant enzyme system in the roots of wild type (WT), *BvM14-SAMDC*-overexpression in WT Arabidopsis (OX). Antioxidant enzyme activities (b to d)under control and salt stress (100 mM NaCl) conditions.

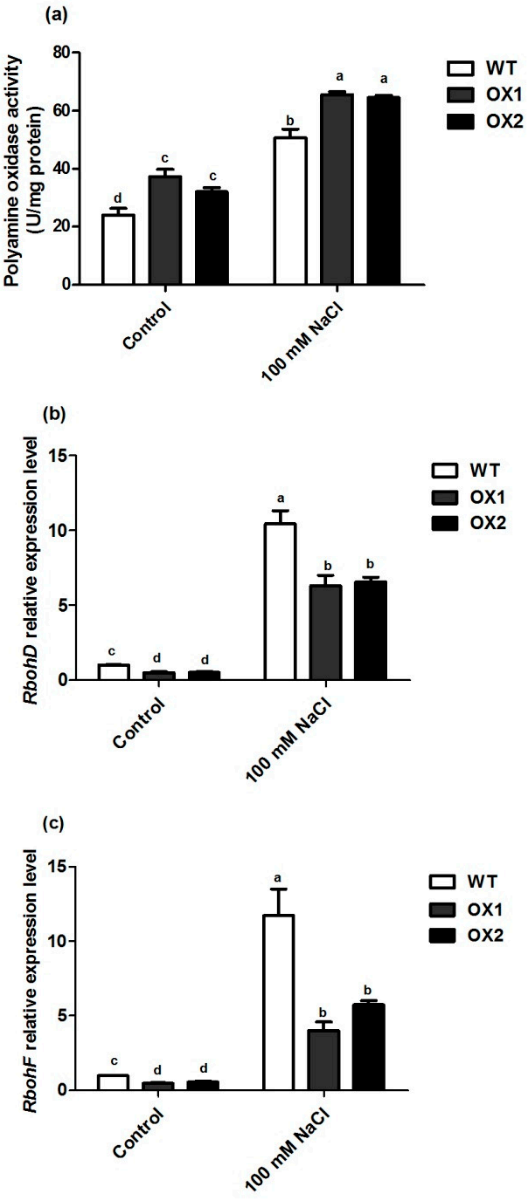

Figure S4. Effects of salt stress on polyamine oxidase (PAO) activity and mRNA levels of *RbohD* and *RbohF* in the roots of wild type (WT) and *BvM14-SAMDC*-overexpression in WT Arabidopsis (OX). (a) PAO activity; (b) mRNA levels of *RbohD*; and (c) mRNA levels of *RbohF* under control and salt stress (100 mM NaCl) conditions.

Table S1 List of the RT-PCR primer

Primer name	Primer sequence
AtRbohD-F（QRT-PCR）	5-TCAGGGACGACTCGGTGG-3
AtRbohD-R（QRT-PCR）	5-GTTTATCGAAACGTTGGTC-3
AtRbohF-F（QRT-PCR）	5-GTTCGATGCATTGAGTAG-3
AtRbohF-R（QRT-PCR）	5-TTTAATCTTGATAGCTTATT-3
BvM14 SAMDC-F（QRT-PCR）	5′-GCTGCTGTGATGACTGAAAG-3′
BvM14 SAMDC-R（QRT-PCR）	5′-TCTTCTGGAATGTGGATGG-3′
18S rRNA-F（QRT-PCR）	5-CCCCAATGGATCCTCGTTA-3
18S rRNA-R（QRT-PCR）	5-TGACGGAGAATTAGGGTTCG-3
Actin-F（Semi-QRT-PCR）	5-ACTCTTAATCAATCCCTCCACC-3
Actin-R（Semi-QRT-PCR）	5-CTGTATGACTGACACCATCACC-3
BvM14 SAMDC-F（Semi-QRT-PCR）	5-TGATGACCCTGTTTATACTCT-3
BvM14 SAMDC-R（Semi-QRT-PCR）	5-TCACTCGGTTCAAAGCAAGC-3
AtSAMDC1-F（Semi-QRT-PCR）	5-AAGTCCCAGCTTGATGAAATTC-3
AtSAMDC1-R（Semi-QRT-PCR）	5-GTGAGGAAAAGGCTGGCCTC-3
